# The prefrontal areas and cerebral hemispheres of the neotropical Cebus apella and its correlations with cognitive processes

**DOI:** 10.1590/S1980-57642010DN40300006

**Published:** 2010

**Authors:** Kellen Christina Malheiros Borges, Jussara Rocha Ferreira, Leonardo Ferreira Caixeta

**Affiliations:** 1 Titular Masters Professor, Federal Institute of Goias, Anápolis GO, Brazil; Anhanguera Educacional University. Anápolis GO, Brazil.; 2 Adjunct Doctor Professor, University of Brasília, Brasília DF, Brazil.; 3 Adjunct Doctor Professor, Behavioral Neurology Unit, Hospital das Clínicas / Federal University of Goias, Goiânia GO, Brazil.

**Keywords:** Cebus apella, brain, cerebral hemispheres, prefrontal area, cognitive processes

## Abstract

The organization of the prefrontal cortex can hold important clues to understanding its functioning. The *Cebus apella* present cerebral particularities and behavioral and cognitive flexibility, possessing abilities that demonstrate an overlap with those of big primates.

**Objectives:**

To provide evidence of correlations between anatomical particularities of the brain areas analyzed and some cognitive abilities previously described in these simians.

**Methods:**

The relative size of the cerebral hemispheres and prefrontal areas (PFA) were measured using a Universal caliper, in 24 hemispheres of C. apella fixed with 10% formaldehyde and kept in 70% alcoholic solution.

**Results:**

Data gathered allowed the calculation of the approximate volume (cm^3^) of the areas under study: right antimere 35.2cm^3^ (±5.3), left antimere 31.3cm^3^ (±5.4) and of the left PFA 6.0cm^3^ (±1.5) and right PFA 6.9cm^3^ (±1.7).

**Conclusions:**

We concluded that the PFA represents about 20% of the cerebral volume of this primate. No significant differences were found in the antimeres in terms of volume and area of the hemispheres and likewise for the PFA. These animals have a proportionally bigger brain than that of other neotropical primates in the literature. This allows us to infer that the frontal lobe of *C. apella* is also larger; possibly related to its maturity and developed cognitive functions indicative of the culture transfers characteristic of this species.

## Abstract (pt)

A organização do córtex pré-frontal pode conter dados importantes para a compreensão de seu funcionamento. Os *Cebus apella* apresentam particularidades cerebrais e flexibilidade comportamental e cognitiva, possuindo habilidades que demonstram destacada sobreposição sobre grandes primatas.

**Objetivos:**

Fornecer subsídios para realização de correlações entre particularidades anatômicas das regiões encefálicas analisadas e algumas habilidades cognitivas já descritas nestes símios.

**Métodos:**

Analisamos o tamanho relativo dos hemisférios cerebrais e da região pré-frontal (RPF), utilizando paquímetro universal, em 24 hemisférios cerebrais de C. apella fixados em formol a 10% e conservados em solução alcoólica a 70%.

**Resultados:**

Os dados obtidos permitiram-nos calcular o volume (cm^3^) aproximado das áreas estudadas: antímero direito 35,2cm^3^ (±5,3), antímero esquerdo 31,3cm^3^ (±5,4) e das RPF esquerda 6,0cm^3^ (±1,5) e a direita 6,9cm^3^ (±1,7).

**Conclusões:**

Concluímos que a RPF representa aproximadamente 20% do volume cerebral deste primata. Não constatamos diferenças significativas nos antímeros em relação aos volumes e às áreas tanto dos hemisférios cerebrais como das RPF. Estes animais apresentaram tamanho cerebral proporcionalmente maior que os demais primatas neotropicais quando comparamos nossos achados com a literatura disponível, permitindo-nos inclusive inferir que há uma grande amplitude do lobo frontal em *C. apella*; possivelmente relacionada à maturidade e às funções cognitivas elaboradas indicativas de transferência de cultura características deste animal.

The extended region of the frontal lobe cortex, located before the premotor areas, is called prefrontal cortex (PFC). In humans and non-human primates the PFC includes all the following areas: rostral, lateral, median and orbit anterior to the front of the premotor cortex.^1^ The prefrontal cortex’s main function is the planning and analysis of consequences of future actions, being related to decision making, judgment, as well as social and ethical behavior.^2-5^ Evidence from human and non-human primates suggests that the prefrontal cortex plays a role in inhibiting control albeit within the domains of specialization.^3^

Maintaining information is also a critical function of the PFC because rule-learning typically involves the formation and association between discrepant events separated by time. Recent events emphasize the relevance of PFC in temporal integration and its role in monitoring and organization of information stored in the brain, an important cognitive ability.^6,7^ The organization of the PFC can reveal important clues as to its function.^8^

The brain has achieved a more elaborate repertoire of behaviors in primates, as a result of its advantageous size and complexity, which culminates in highly sophisticated cultural behaviors in humans such as language, the usage of tools and social learning.^9,10^

The *Cebus apella* are animals weighing about 3kg that are noted for their motor and cognitive abilities. The usage of objects is very common and constantly reported within wild *Cebus apella* as opposed to bred ones.^11-14^ Similarly to chimpanzees, there are reports of the usage of stone tools by wild *Cebus apella* to crack nuts and to split open Jerivá coconuts (*Syagrus romanzoffiana*) or in induced manner, of the use of sticks to catch food from tubes and extract honey-like substances from holes in a box.^12-18^

The *C. apella*, usually considered less suitable than big primates for certain research studies, have recently drawn the interest of scientists due to their high cerebral particularities and behavioral and cognitive flexibility, possessing abilities that demonstrate overlaps with those of big primates.^18-22^

## Methods

A total of 24 hemispheres of *Cebus apella* (consisting of 12 left antimeres and 12 right antimeres) preserved in a 10% formaldehyde solution were used in this study. These specimens were provided by the Surgery Department of the School of Veterinary and Animal Science of the University of São Paulo. They had been used in previous studies and kept for further use so as to avoid the unnecessary sacrifice of animal lives, in conformance with international norms of bioethics and animal wellbeing. The study considered the weight of the cerebral hemispheres and the relationship of the volume of the prefrontal areas compared to the whole brain from the same animal. This study was conducted according to the rules of ethics in animal research.

In order to compare data obtained from *C. apella*, measurements were made of human hemispheres and the prefrontal regions using 5 brains (consisting of 5 left antimeres and 5 right antimeres) from the Department of Morphology and Anatomy of Anhanguera Educational University. To perform the measurement in humans, we followed guidelines concerning the delimitation of the prefrontal region described by Barbas (1995).

### Prefrontal area volume analysis

A number of proposals have been made regarding the possible correspondences between areas of the human and macaque frontal lobes.^1,4,23-28^ According to several studies on the prefrontal cortex of other primates^4,28-31^ the delimitation of the prefrontal cortex in its lateral surface is defined as the portion anterior to the arcuate sulcus, while for the medial surface, a reasonable delimitation for the PFC can be defined as all portions of the frontal cortex anterior to the genu of the corpus callosum, in a plane perpendicular to the line connecting the anterior and posterior commissures.

Because it is difficult to delimit the prefrontal cortex unambiguously using gross sulcal landmarks, it has been argued that definitive comparative quantitative analysis would require extensive detailed cytoarchitectural studies that, because of their expense, are unlikely to be carried out in the near future.^28^

**Figure 1:**
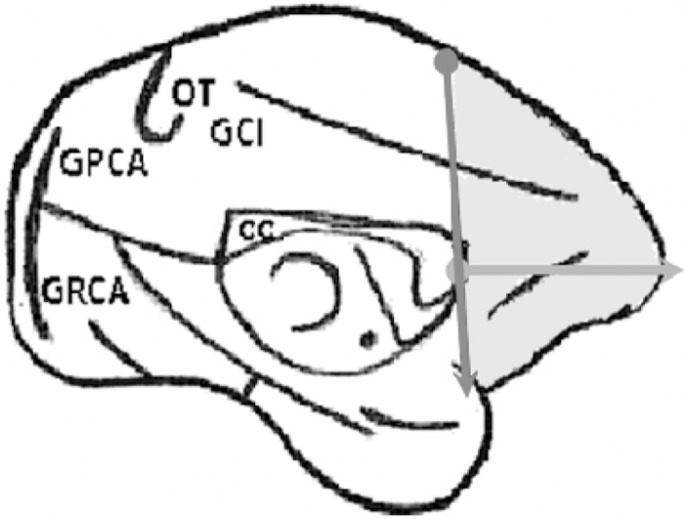
Diagram of brain of Cebus apella (medial view), demonstrating the limits of the measurements of the prefrontal area. GPCA: paracalcarine gyrus; GRCA: retrocalcarine gyrus, OT: occipitotemporal gyrus; GCI: cingulate gyrus; GR: rostral gyrus; CC: corpus callosum.

**Figure 2:**
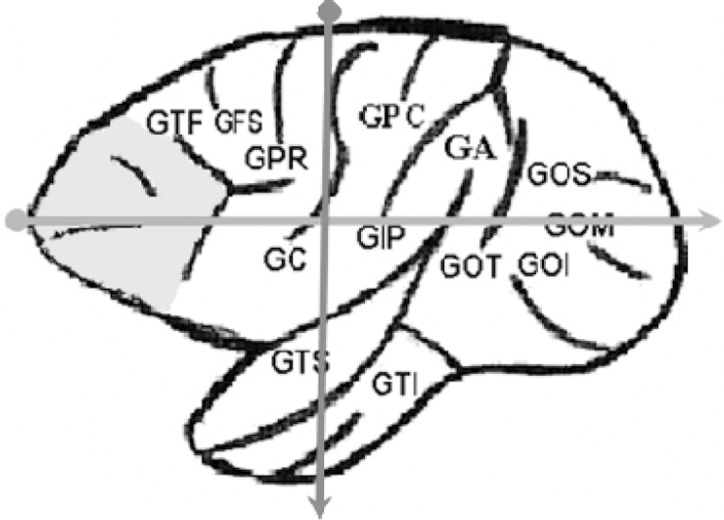
Diagram of brain of Cebus apella (side view), demonstrating the limits of the measurements of the cerebral hemisphere. FG: frontal gyrus; GFM: middle frontal gyrus; GTF: triangular frontal gyrus; GFS: superior frontal gyrus; GPR: pre-central gyrus; GC: central gyrus; GPC: postcentral gyrus; GIP: intraparietal gyrus; GA: gyrus angular; GOT: transverse occipital gyrus; GOS: superior occipital gyrus; GOM: middle occipital gyrus; GOI: inferior occipital gyrus; GTS: superior temporal gyrus; GTI: inferior temporal gyrus.

Using a Universal caliper (scale of 0-300 mm, resolution 0.05 mm, Digimess, Rio de Janeiro, Brazil) the prefrontal regions and the cerebral hemispheres of the specimens were measured (Figures 1 and 2).

To obtain the estimated volume of each prefrontal area the following distances were taken into account:

- The anterior part of the arcuate sulcus up to the anterior pole of the frontal lobe (length in cm);
- From the longitudinal fissure of the brain to the most prominent portion of the lateral surface of the frontal lobe (width in cm);
- From the higher pole of the frontal lobe to the lower pole (height in cm).

The values obtained through these measurements (in centimeters) when multiplied together yielded the approximate volume in cm^3^ of each prefrontal cortex. The same process allowed the gathering of values for analysis of the cerebral hemispheres, where the following measurements were made:

- From the anterior most prominent region of the frontal pole to the posterior region of the occipital lobe (length in cm);
- From the longitudinal fissure of the brain to the most prominent median lateral surface (width in cm);
- From the upper top of the brain, in the precentral gyrus to the lower portion of the temporal lobe (height in cm).

All the values of these measurements (in centimeters) when multiplied together yielded the approximate volumes (cm^3^) of the cerebral hemispheres.

The *t* test (P<0.05) was used to compare the volumes of the right and left cerebral hemispheres and to compare the volumes of the right and left prefrontal areas. The range of variation in the measures of the right and left volumes (for the cerebral hemispheres as well as prefrontal areas) were analyzed using the Coefficient of Variation (CV).

## Results

### Volume of the right and left cerebral hemispheres

With the purpose of calculating volume in cubic centimeters of the cerebral hemispheres, the lengths, heights and widths (according to the methodology proposed) were measured. These values and measures can be found in Table 1.

**Table 1:** Measurements of dimensions of cerebral hemispheres (*Cebus apella*) for calculations of volume, mean and standard deviation in total sample.

Case number			CH length (cm)			CH height (cm)			CH width (cm)			CH volume (cm^3^)	
**ra**	**la**		**ra**	**la**		**ra**	**la**		**ra**	**la**		**ra**	**la**
4	1		5.1	5.3		3.3	3.3		1.9	1.7		32.0	29.7
9	2		5.7	5.2		3.9	3.2		1.9	1.7		42.2	28.3
10	3		5.5	5.0		3.6	3.0		1.8	1.7		35.6	25.5
12	5		5.8	5.9		3.6	3.5		1.8	2.0		37.6	41.3
13	6		5.2	5.3		3.5	3.3		1.7	1.9		30.9	33.2
15	7		5.3	5.4		3.2	3.6		1.8	1.8		30.5	35.0
17	8		5.1	5.5		3.2	3.5		2.1	2.0		34.3	38.5
19	11		5.5	5.3		3.7	3.1		1.9	1.6		38.7	26.3
21	14		5.6	5.2		3.2	3.1		1.7	2.2		30.5	35.5
22	16		6.0	5.2		3.9	3.3		2.0	1.8		46.8	30.9
23	18		5.8	4.8		3.0	3.0		1.7	1.7		29.6	24.5
24	20		5.2	5.0		3.4	3.4		1.9	1.6		33.6	27.2
Mean (SD)			5.5 (±0.3)	5.3 (±0.3)		3.5 (±0.3)	3.3 (±0.2)		1.9 (±0.1)	1.8 (±0.2)		35.2 (±5.3)	31.3 (±5.4)

SD: standard deviation; CH: cerebral hemisphere; ra: right antimere; la: left antimere.

Table 2 shows the estimated volume of the right and left prefrontal area, respectively. The mean estimated volume of the cerebral hemispheres was 33.3 cm^3^ (cerebral volume of 66.6 cm^3^) with a standard deviation of ±5.4for the left and ±5.3 for the right hemispheres. The mean estimated volume of the prefrontal area (PFA) was of 6.4 cm^3^ (total volume of 12.8 cm^3^) with a standard deviation of ±1.5 for the left antimeres and ±1.7for the right antimeres.

**Table 2:** Measurements of dimensions of prefrontal areas (*Cebus apella*) for calculations of volume, mean and standard deviation in total sample.

Case number			PFA length (cm)			PFA height (cm)			PFA width (cm)			PFA volume (cm^3^)	
**ra**	**la**		**ra**	**la**		**ra**	**la**		**ra**	**la**		**ra**	**la**
4	1		1.6	2.0		2.3	2.3		2.1	1.3		7.7	6.0
9	2		2.3	1.5		2.7	2.4		1.6	1.2		9.9	4.3
10	3		2.0	2.2		2.5	2.2		1.2	1.0		6.0	4.8
12	5		2.6	2.5		2.5	2.5		1.4	1.3		9.1	8.1
13	6		2.1	1.9		2.3	2.1		1.3	1.1		6.3	4.4
15	7		2.1	2.0		2.3	2.5		1.1	1.3		5.3	6.5
17	8		1.7	2.0		2.0	2.3		1.3	1.7		4.4	7.8
19	11		2.3	1.9		2.5	2.3		1.5	1.1		8.6	4.8
21	14		2.3	2.0		2.3	2.2		1.1	1.4		5.8	6.2
22	16		2.1	2.2		2.8	2.3		1.3	1.6		7.6	8.1
23	18		1.9	1.7		2.0	2.0		1.5	1.3		5.7	4.4
24	20		2.0	2.2		2.0	2.2		1.5	1.3		6.0	6.3
Mean (SD)			2.1 (±0.3)	2.0 (±0.3)		2.4 (±0.3)	2.3 (±0.1)		1.4 (±0.3)	1.3 (±0.2)		6.9 (±1.7)	6.0 (±1.5)

SD: standard deviation; PFA: prefrontal area; ra: right antimere; la: left antimere.

The analysis of mean cerebral volume (66.6 cm^3^) compared to mean estimated volume of the prefrontal area (12.8 cm^3^), allows us to infer that in these animals the prefrontal area represented approximately 19.21% of the total cerebral volume.

Comparing the volumes of the right and left cerebral hemispheres with the t test, no statistical significant difference was observed between the two sides (P>0.05). Similarly, no difference was found in comparisons of the volumes of the left and right prefrontal areas.

The variation range of the measurements of the left and right antimeres for the cerebral hemispheres and the prefrontal areas was very similar in terms of Coefficient of Variation (CV). The CV for the left cerebral hemisphere was 17% and the right was 15%. The CV in relation to the left prefrontal area was 24% and for the right was 25%.

Tables 3 and 4 contain measurements of dimensions of human cerebral hemispheres and prefrontal areas, respectively.

**Table 3:** Measurements of dimensions of human cerebral hemispheres for calculations of volume, mean and standard deviation in total sample.

Case number			CH length (cm)			CH height (cm)			CH width (cm)			CH volume (cm^3^)	
**ra**	**la**		**ra**	**la**		**ra**	**la**		**ra**	**la**		**ra**	**La**
1	1		16.5	17.1		9.0	10.6		6.2	7.2		920.7	1305.1
2	2		16.3	15.7		9.2	9.8		6.6	6.5		989.7	1000.1
3	3		16.1	16.8		7.4	7.6		6.3	6.6		750.6	842.7
4	4		16.8	17.2		8.5	8.9		6.3	5.8		899.6	887.9
5	5		15.9	16.3		9.3	8.8		6.3	5.4		931.6	774.6
Mean (SD)			16.3 (±0.3)	16.6 (±0.6)		8.7 (±0.8)	9.1 (±1.1)		6.3 (±0.2)	6.3 (±0.7)		898.4 (±89.2)	962.1 (±208.6)

SD: standard deviation; CH: cerebral hemisphere; ra: right antimere; la: left antimere.

**Table 4:** Measurements of dimensions of human prefrontal areas for calculation of volume, mean and standard deviation in total sample.

Case number			PFA length (cm)			PFA height (cm)			PFA width (cm)			PFA volume (cm^3^)	
**ra**	**la**		**ra**	**la**		**ra**	**la**		**ra**	**la**		**ra**	**la**
1	1		5.4	5.5		7.6	8.0		5.8	4.3		238.0	189.2
2	2		5.3	5.7		6.8	6.8		5.9	4.5		212.6	174.4
3	3		5.1	5.7		6.3	6.5		5.0	5.3		160.7	196.4
4	4		5.3	5.9		7.4	7.0		5.7	5.0		223.6	206.5
5	5		5.3	4.9		6.9	7.0		5.2	4.5		190.2	154.4
Mean (SD)			5.3 (±0.1)	5.5 (±0.4)		7.0 (±0.5)	7.1 (±0.6)		5.5 (±0.4)	4.7 (±0.4)		205.0 (±30.3)	184.2 (±20.4)

SD: standard deviation; PFA: prefrontal area; ra: right antimere; la: left antimere.

The mean estimated volume of the human cerebral hemispheres was 930.3 cm^3^ (cerebral volume of 1860.5 cm^3^) with a standard deviation of ±208.6 for the left and ±89.2 for the right hemispheres. The mean estimated volume of the prefrontal area (PFA) was 194.6 cm^3^ (total volume of 389.2 cm^3^), with standard deviation of ±20.4 for the left antimeres and ±30.3 for the right antimeres.

The analysis of mean cerebral volume (1860.5 cm^3^) compared to mean volume estimated from the prefrontal area (389.2cm ^3^), allows us to infer that in humans the prefrontal area represents approximately 20.92% of total cerebral volume.

Comparing the volumes of the right and left human cerebral hemispheres with the *t* test, no statistical significant difference was found between them (P>0.05). Similarly, no significant difference was found on comparison of the volumes of human left and right prefrontal areas.

## Discussion

The analyses of the volumes of cerebral hemispheres and of prefrontal volumes of the *Cebus apella* showed no relevant differences between left and right antimeres, a result validated by the statistical test. The test showed that there is a probability of both regions developing proportionally, justifying no hemispherical asymmetry.

In this study the method of measurement of the cerebral hemispheres allowed us to gather data for the linear measurements of the prefrontal area, as well as those of the cerebral hemisphere, without damaging the specimens thereby allowing for their use in future studies.

Comparisons of the mean brain volumes (66.6 cm^3^ in C. apella and 1860.5 cm^3^ in humans) and means volumes of prefrontal regions (12.8 cm^3^ in *C. apella* and 389.2 cm^3^ in humans) found in our study revealed that the prefrontal region in *C. apella* presented a similarly significant volume (19.21% of total brain volume) to humans (20.92% of total brain volume). This demonstrates that these primates indeed possess a well-developed prefrontal area, corroborating data reported in other studies involving capuchin monkeys. There is evidence that the anatomical organization of the prefrontal area allows the possibility of correlation between the development of this region in the *C. apella* with the advanced cognitive processes governed by the PFA.^1,13,15,18,22^

Comparison of mean brain volumes using the *t* test showed a statistical significant difference (P<0.01) between the data obtained in this study and data obtained by Schoenemann et al. This demonstrates real differences in methodologies employed for examinations. However, it is pertinent to note that discrepancies in neuroanatomical studies could stem from difficulty in accurately defining the prefrontal region in different mammals.^32^

The methodology used in the present study differed to that used by Schoenemann et al. These authors analyzed brains of several primates, including *C. apella* and humans, using magnetic resonance imaging, a modern technique of analysis. In our study, we needed to maintain the integrity of the specimen for analysis of the process of intra-hemispheric association, observed after dissection (by the method of Klingler). Therefore, we chose to use a technique of measuring the hemispheres with the aid of calipers, which enabled us to gather data on the linear measurements of the prefrontal region as well as the full hemisphere, without damaging the specimen.

We recognize that our technique using the calipers has limitations for analysis of brain surface irregularities, yet the findings were relevant because the results for both the estimated volume of the prefrontal region as well as the total estimated volume were in percentages, i.e. the volume of the prefrontal region of each brain examined was calculated based on estimates.

Although relative brain size is difficult to quantify and correlate with behavior, the increased relative brain size is usually accompanied by increased complexity of foraging.^33^ A previous study suggested that the complexity of brain connections had a fundamental role in the evolution of the brain and that changes in the relative proportions of different parts of the brain probably allowed behavioral adaptation.^28^

The *Cebus apella* have a encephalization degree which is greater than a variety of other primates including those considered philogenetically closer to man.^34,35^ Studies clearly show a higher development of the prefrontal areas of these primates of the New World, considering the white matter and gray matter of the brain.^28^ These animals are intelligent with a manipulative extractivist style, using a foraging mechanism derived from a potential for cultural variation in natural and artificial environments, a feature which may be attributed to its cerebral development.^36,37^ Significant cultural aspects are evident, such as those described in learning how to break nuts, as well as in cooperation among animals to obtain food.^16,21,38^

In wild and artificial environments the *C. apella* has been shown to use stone tools with ease, to break nuts and extract the pulp, behavior hitherto considered to be a characteristic of only certain chimpanzee groups.^11,13,14,18^ The *C. apella* not only selected the right tool, but modified it to make it more efficient^39^ as some chimpanzees do, for separating leaves from stems to get ants. These data, and other indications of the usage of tools and socially adequate behaviors among monkeys, suggest the species might have a rudimentary form of culture, probably developed through evolutive changes in the prefrontal area of these primates.

It is known that primates possess substantially enlarged association areas especially in the frontal area. The managing of tools, according to recent studies in humans, is associated to the medium frontal and inferior frontal gyrus. These regions help in the integration between adequate manipulation of objects and their function. This is achieved by the frontal cortex coding the appropriate sequence of hand movements so that the correct handling of a tool occurs.^40^

It is legitimate to conclude that the evolutive expansion of the prefrontal association cortex is related to the evolution of the cognitive functions. Some examples of this observed in the *C. apella* are its creativity in the usage of fruit as bait^20^ and the ability for image interpretation and its related behaviors (theory of mind).^22^ Studying primate non-human species in a comparative and multifaceted manner helps to evaluate the neural functions in different groups. Understanding the pressures that led to these cognitive abilities may be of fundamental importance in acknowledging the organ responsible for controlling these abilities: the brain.
